# Characterization of Canine Influenza Virus A (H3N2) Circulating in Dogs in China from 2016 to 2018

**DOI:** 10.3390/v13112279

**Published:** 2021-11-15

**Authors:** Yuanguo Li, Xinghai Zhang, Yuxiu Liu, Ye Feng, Tiecheng Wang, Ye Ge, Yunyi Kong, Hongyu Sun, Haiyang Xiang, Bo Zhou, Shushan Fang, Qing Xia, Xinyu Hu, Weiyang Sun, Xuefeng Wang, Keyin Meng, Chaoxiang Lv, Entao Li, Xianzhu Xia, Hongbin He, Yuwei Gao, Ningyi Jin

**Affiliations:** 1 College of Veterinary Medicine, Jilin University, Changchun 130062, China; liyuanguo0520@163.com; 2 Key Laboratory of Jilin Province for Zoonosis Prevention and Control, Changchun Veterinary Research Institute, Chinese Academy of Agricultural Sciences, Changchun 130122, China; Xinghaizhang2016@outlook.com (X.Z.); fengye621@126.com (Y.F.); wgcha@163.com (T.W.); qfkongyy@163.com (Y.K.); haiyang198710@163.com (H.X.); hottank3210@126.com (B.Z.); fangshushan2021@163.com (S.F.); q.xia@novo-biotech.com (Q.X.); huxinyu180@163.com (X.H.); sunweiyang1987@163.com (W.S.); xuefeng_wangNUDT@outlook.com (X.W.); mengkeyin@126.com (K.M.); lucx773@nenu.edu.cn (C.L.); liet0706@163.com (E.L.); xiaxzh@cae.cn (X.X.); 3 Wuhan Institute of Virology, Chinese Academy of Sciences, Wuhan 430071, China; 4 National Research Center for Veterinary Medicine, Luoyang 471003, China; qingqingyuxiu@hotmail.com; 5 College of Coastal Agricultural Sciences, Guangdong Ocean University, Zhanjiang 524088, China; geye_perfect@126.com; 6 College of Basic Medical Sciences, Jilin Medical University, Jilin 132013, China; sunhongyu20@163.com; 7 College of Life Science, Shandong Normal University, Jinan 250014, China

**Keywords:** canine influenza virus, evolution, serological surveillance, transmission

## Abstract

Avian H3N2 influenza virus follows cross-host transmission and has spread among dogs in Asia since 2005. After 2015–2016, a new H3N2 subtype canine influenza epidemic occurred in dogs in North America and Asia. The disease prevalence was assessed by virological and serological surveillance in dogs in China. Herein, five H3N2 canine influenza virus (CIV) strains were isolated from 1185 Chinese canine respiratory disease samples in 2017–2018; these strains were on the evolutionary branch of the North American CIVs after 2016 and genetically far from the classical canine H3N2 strain discovered in China before 2016. Serological surveillance showed an HI antibody positive rate of 6.68%. H3N2 was prevalent in the coastal areas and northeastern regions of China. In 2018, it became the primary epidemic strain in the country. The QK01 strain of H3N2 showed high efficiency in transmission among dogs through respiratory droplets. Nevertheless, the virus only replicated in the upper respiratory tract and exhibited low pathogenicity in mice. Furthermore, highly efficient transmission by direct contact other than respiratory droplet transmission was found in a guinea pig model. The low-level replication in avian species other than ducks could not facilitate contact and airborne transmission in chickens. The current results indicated that a novel H3N2 virus has become a predominant epidemic strain in dogs in China since 2016 and acquired highly efficient transmissibility but could not be replicated in avian species. Thus, further monitoring is required for designing optimal immunoprophylactic tools for dogs and estimating the zoonotic risk of CIV in China.

## 1. Introduction

Influenza A viruses (IAVs) are segmented single-stranded RNA (ssRNA) viruses belonging to *Orthomyxoviridae* that have a complex natural history with a wide range of host species [1,2]. Based on the combination of hemagglutinin (HA) and neuraminidase (NA) glycoproteins, IAVs could be divided into different subtypes consisting of 18 HA and 11 NA subtype viruses [3]. Wild waterfowl are the major natural reservoir hosts. Among them are common viruses belonging to 16 HA (H1–H16) and 9 NA (N1–N9) serotypes [4,5]. Occasionally, avian influenza viruses can cross the species barrier and acquire the ability to infect other animals, including swine [6], equines [7], seals [8], dogs [9], wild and domestic cats [10], civets [11], nonhuman primates [12], chinstrap penguins [13], martens, and humans [14,15,16]. Some biological factors, such as the host cell receptor (sialic acid) and its linkages, play critical roles in cross-species transmission [17]. The canine respiratory tract expresses both α-2,3- and α-2,6-linked sialic acid receptors, which provide the prerequisite for avian influenza virus infection in dogs [18].

In 2005–2006, the avian-origin H3N2 was first reported in canines in Asia, including South Korea and China, and later acquired the capability of dog-to-dog transmission [19,20]. The H3N2 canine influenza virus (CIV) is still prevalent in several provinces across China according to virological surveillance and seroepidemiological investigation carried out in the past decade [21,22]. CIV has undergone adaptive changes to stably circulate in the Asian canine population during the decade since its discovery [23]. In February 2015, the H3N2 CIV was firstly introduced in the USA, causing an epidemic in dogs [24]. The evolutionary processes of H3N2 CIV encompassed rapid fade-out, followed by multiple incursions and recurrent epidemics in the USA in 2015–2017 [25]. The CIV A (H3N2) clade with antigenic variation occurred in China in 2016–2017 [26]. This clade could have originated from H3N2 CIVs circulating in South Korea or the USA rather than from ancestral H3N2 CIVs from China.

In order to determine the circulation dynamics in China, virological surveillance and seroepidemiological investigation of CIVs were carried out in this study. Chinese families are exposed to a large number of pet dogs that may serve as intermediate hosts for transmitting the influenza virus to humans.

With the increasing number of pet dogs in China, whether the canine could serve as an intermediate host for transmitting the influenza virus to humans is a major concern. The reassortment events between CIVs and other influenza viruses, including the H5N1 avian virus and the human H1N1 pandemic virus, have occurred [23,27]. Several CIV genotypes were generated via reassortment events in canine hosts, indicating the capacity of dogs to serve as “mixing vessels” [28]. Additionally, CIVs might pose a threat to public health for their ability to spread efficiently among mammals via respiratory droplets [29]. However, the extent of infectivity in dogs and onward transmission among mammals remains unknown with respect to these new clade viruses. In this study, we isolated five strains of H3N2 influenza virus (0.42%) from 1185 swab samples and assessed one of them in terms of the replication and transmission of four from five isolates CIV H3N2 in guinea pigs and beagles.

## 2. Materials and Methods

### 2.1. Virological Investigation

A total of 1185 nasal and throat swabs were collected from dogs with respiratory symptoms in veterinary clinics or kennels in Jilin, Henan, and Guangdong provinces in China to monitor the H3N2 CIV epidemic and virus evolution in January 2017–May 2019. Sample processing and virus isolation were conducted as described previously [30]. Embryonated specific pathogen-free (SPF) eggs (9–11 days old) were inoculated with swab samples for virus isolation. The hemagglutination test was performed to determine the presence of the virus. RNA was directly extracted from the allantois fluid for whole genome amplification by RT-PCR. Primers (synthesized by Comate Bioscience Co., Ltd., Changchun, China) were designed for each H3N2 CIV fragment to amplify the whole genome, according to the sequence published by GenBank (Supplementary Table S1).

### 2.2. Phylogenetic Analysis

To characterize the evolution of CIV H3N2, we sequenced the full genome of five strains and performed genetic analyses using H3N2 virus sequences of human, avian, and canine species from GenBank (www.ncbi.nlm.nih.gov accessed on 5 July 2021) and the GISAID (www.gisaid.org accessed on on 5 July 2021) database. Consensus sequence editing, alignment, and phylogenetic analysis were performed with Mega7.0. Total sequence alignment lengths (in nucleotides (nt)) were as follows: PB2, 2122; PB1, 2134; PA, 2048; HA, 1598; NP, 1394; NA, 1334; M1, 727; NS1, 655. Phylogenetic analyses were carried out for all eight gene segments using the maximum-likelihood (ML) method with 1000 bootstrap replicates, the general time-reversible (GTR) substitution model, and gamma-distributed rate variation among sites.

### 2.3. Seroepidemiological Investigation

A total of 3197 serum samples fom clinically healthy pet dogs were collected across 27 provinces in China, of which 913 were collected from January to December in 2017 and 2284 were collected from January to December in 2018. In addition, 208 serum samples were collected from pet dogs with clinical symptoms of respiratory disease in veterinary clinics in 2018, 130 serum samples were collected from stray dogs in Jilin in 2017, and 44 serum samples were collected from Chinese rural dogs in Shannxi in 2018. The hemagglutination inhibition (HI) test was used to detect the serum samples. The test was performed according to procedures recommended by the World Organization for Animal Health (OIE) (https://www.oie.int/fileadmin/Home/eng/Health_standards/tahm/3.03.04_AI.pdf accessed on 25 May 2021). Briefly, 25 μL of serial two-fold dilutions of serum samples were mixed with four units of hemagglutination titer of the virus (A/canine/Jilin/QiaoKe01/2017 (H3N2) CIV strain (GenBank accession no. MZ948848-MZ948855)) in V-shaped microtiter plates and incubated at room temperature for 30 min. Then, 25 μL of 1% chicken red blood cells (RBCs) were added to each well and incubated at room temperature for 40 min. The HI titer was expressed as the reciprocal of the highest serum dilution that completely inhibited the hemagglutination of the virus. Serum samples with up to 8 antibody titers were considered positive. The animal experiment was approved by the Institutional Review Board (or Ethics Committee) of Key Laboratory of Jilin Province for Zoonosis Prevention and Control (IACUC no. AMMS-11-2017-001, approved on 12 May 2017).

### 2.4. Replication and Transmission in Beagles

A total of 11 beagles (4 months old) that tested negative for serological antibody against H3N2 CIVs were chosen from the Rui man dog training field, Shenbei New Area (Shenyang, China), and assigned randomly to four groups, including inoculated (*n* = 4), contact (*n* = 3), exposed (*n* = 3), and control (*n* = 1) groups. Dogs in the inoculated group were anesthetized with ketamine and inoculated intranasally with 10^7^ EID_50_ (50% egg infections dose) in a volume of 1 mL and placed into the contact (same cage) and exposed (1 m distance) groups after 24 h. All dogs were monitored daily for temperature, physical activities, and nasal/throat swabs. At 3 days post-infection (dpi), one dog from the inoculated group was euthanized, and each lung lobe (upper left, lower left, middle, upper right, and lower right), lung lymph nodes, spleen, intestine, mesenteric lymph nodes, and trachea were taken for hematoxylin–eosin (H&E) staining and immunohistochemistry (IHC) with mouse antiserum against the QK01 virus and goat anti-mouse IgG as the secondary antibody (Abcam, MD, USA). The nasal and throat swabs of each dog were mixed to determine the EID_50_ by injection of chicken embryos. The venous blood of each dog was collected to determine HI antibody levels at 14, 21, 28, and 35 dpi.

### 2.5. Pathogenesis in Mice

A total of 30 BALB/c mice (16–18 g, female) were purchased from the Beijing Vital River Laboratory (Beijing, China) and randomly divided into six groups. The virus solution was diluted to a range of 10^2^–10^6^ EID_50_/50 μL. All mice were lightly anesthetized with isoflurane and inoculated intranasally (i.n.) with the diluted virus solutions in a volume of 50 μL, and the control group was inoculated with an equivalent volume of phosphate-buffered saline (PBS). At 3 dpi, three mice in the 10^6^ EID_50_ group were sacrificed, and organs (brain, intestine, spleen, kidney, brain, nasal turbinate, and lung) were collected for viral RNA load analysis. The animal body weight was monitored daily until 14 dpi.

### 2.6. Replication and Transmission in Guinea Pig

Totally 12 Hartley female guinea pigs (350–380 g; Vital River Laboratories, Beijing, China) were used to assess the transmission capacity of the virus, as described previously (13). All animal experiments were carried out in special cages. Each cage had two independent spaces on the left and right sides separated by 5 cm. The animals were randomly divided into three groups: inoculated (*n* = 3), contact (*n* = 3), and exposed (*n* = 3). The six guinea pigs in the inoculated group were anesthetized with ketamine and inoculated intranasally with 2 × 10^6^ EID_50_ in a volume of 200 μL; of these, three guinea pigs were placed on the left side of three cages individually. After 24 h, each of these animals was placed in a cage with one uninfected guinea pig. The other uninfected guinea pig was placed in a nearby cage (5 cm apart) as the exposed group (Supplementary Figure S1). The nasal wash was collected daily to detect the shedding of the virus. Blood serum was obtained at 7, 14, and 21 dpi to determine HI antibody levels, and the seroconversion of animals was confirmed by the HI test. In the same way, 3 other guinea pigs were inoculated intranasally (2 × 10^6^ EID_50_, 200 μL). Then, they were euthanized on days 3, 5, and 7 post-inoculation individually, and the lungs and tracheas were collected for H&E and IHC staining to observe pathological changes and virus replication.

### 2.7. Infection of Chickens and Ducks

In order to assess whether the virus can infect poultry after long-term adaptation in dogs, we chose SPF chickens and domestic ducks for infection. All animals were seronegative for H3N2 CIV. A total of 15 SPF chickens (4-week-old females) were purchased from Harbin Weike Biotechnology Co., Ltd. (Harbin, China) and randomly divided into four groups: inoculated (*n* = 7), contact (*n* = 3), exposed (*n* = 3), and control (*n* = 2). Chickens in the inoculated group were infected intranasally with 3 × 10^6^ EID_50_/0.1 mL and placed into the contact (same cage) and exposed (0.5 m distance) groups after 24 h. Throat and cloacal swabs were collected daily to detect virus titers in 9-day-old chicken embryos. Venous blood samples in all groups were collected to determine HI antibody levels on 14 and 21 dpi. Furthermore, 11 domestic ducks (9 weeks old) were purchased from Changchun Hao Yi animal farm (Changchun, China) and randomly divided into four groups: inoculated (*n* = 3), contact (*n* = 3), exposed (*n* = 3), and control (*n* = 2). The experimental protocol was the same as the above chicken experiment.

## 3. Results

### 3.1. Virological Investigation

Five strains of the H3N2 influenza virus (0.42%) were isolated from 1185 swab samples from canines. Swab samples from dogs were collected in animal hospitals in four provinces, including Jilin (*n* = 397), Liaoning (*n* = 616), Henan (*n* = 24), Jiangsu (*n* = 50), and Guangdong (*n* = 98) in 2014–2019. The 1185 swab samples consisted of 5 positive nucleic acids across Jilin (1), Henan (3), and Guangdong (2).

The isolated strains in this study were termed A/canine/Jilin/QiaoKe01/2017 (QK01(H3N2)), A/canine/Henan/L02/2018 (L02(H3N2)), A/canine/Henan/L03/2018 (L03(H3N2)), A/canine/Henan/L06/2018 (L06(H3N2)), and A/canine/Guangdong/GY01/2018 (GY01(H3N2)). The sequences of these isolates were submitted to GenBank at NCBI (GenBank accession MZ948848-MZ948855 and OK354170-OK354201), and A/canine/Jilin/QiaoKe01/2017 (QK01(H3N2)) was selected for further characterization.

### 3.2. Phylogenetic Analysis

**Figure 1:**
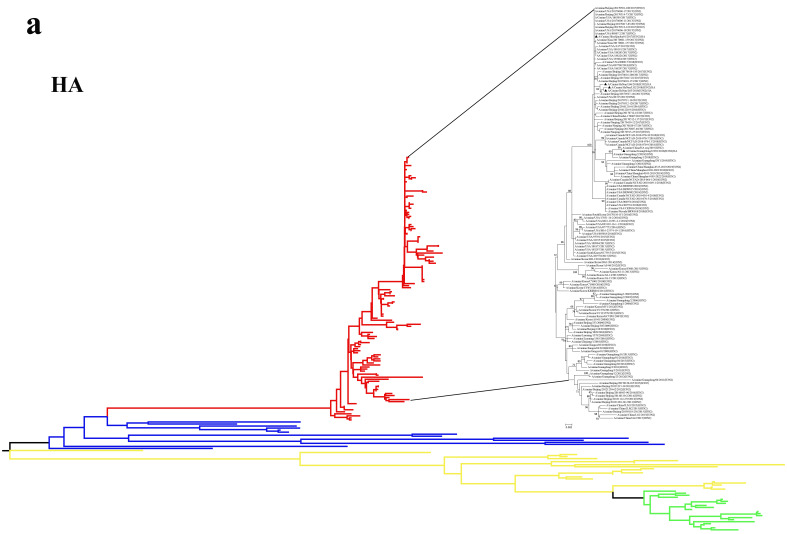

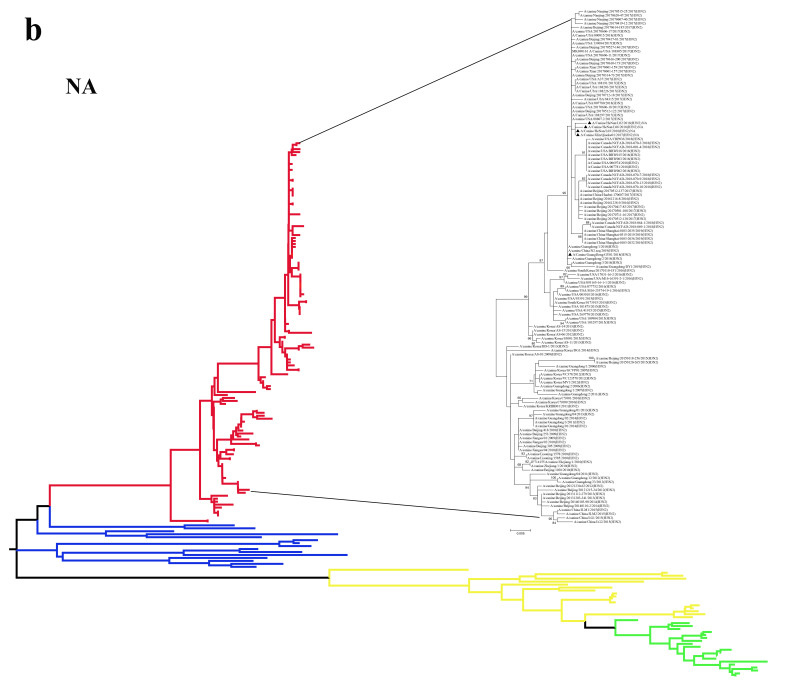
Phylogenetic tree of HA and NA sequences of H3N2: (**a**) phylogenetic tree of HA sequences of H3N2. (**b**) Phylogenetic tree of NA sequences of H3N2. Red, blue, yellow, and green branches represent isolates from dogs, avian, swine, and humans, respectively. Black circles represent the strains in this study.

In order to determine the evolution of H3N2 CIV in 2017–2019, we performed a phylogenetic analysis of eight segments. Each genome segment of the five isolated H3N2 CIVs shared a high nucleotide sequence identity. The phylogenetic analysis of HA and NA indicated that H3N2 strains isolated from dogs are independent evolutionary branches in the phylogenetic tree, which is far from H3N2 in humans and swine (Figure 1). The phylogenetic analysis of HA and NA showed that the GY01 and H3N2 strains found in Guangdong dogs in 18 years clustered together and formed an evolutionary branch, indicating that the GY01 strain harbored an H3N2 CIV group prevalent in Guangdong. However, it was distal to the canine H3N2 strain reported in Guangdong from 2010 to 2015, which indicated that the strains found in recent years in this province were not of the previous epidemic group but a new H3N2 group. The group also had a close evolutionary correlation with the H3N2 strain found in Nanjing dogs in 2017. The L02, L03, and L06 strains collected from Henan Province were clustered together and were in a sizeable evolutionary branch with QK01. The evolutionary branch consisted of many 2017–2018 strains of canine H3N2 reported in the USA and Beijing in the past two years, far from the H3N2 strain reported in mainland China before 2016. This indicated that the canine H3N2 strain reported in China in recent years is not the domestic epidemic strain known before 2015. It may have spread from the USA to China and further spread in China through pet trade and other channels. The phylogenetic analysis of M, NP, NS, PA, PB1, and PB2 of H3N2 showed that the six fragments were consistent with HA and NA in the evolutionary correlation, i.e., the isolated strains (L02, L03, and L06) and QK01 isolated from Jilin dogs were closer to H3N2 strains isolated from Beijing and the USA in 2017–2018; the GY01 strain in Guangdong was more similar to the local endemic strain in the province from 2017 to 2018 (Supplementary Figure S2). Evolutionary analyses showed that all six genes of these five viruses were in the same cluster with the H3N2 CIV reported earlier in 2017. Furthermore, no reassortment was detected among the five viruses via segment phylogeny (Supplementary Figure S2).

Phylogenetic tree of H3N2 CIV internal sequences.

In this study, no virus belonging to the clade before 2016 was detected, suggesting that the new antigenic variation lineage arisen in 2017 was dominant in the dog population.

### 3.3. Seroepidemiological Investigation

**Figure 2:**
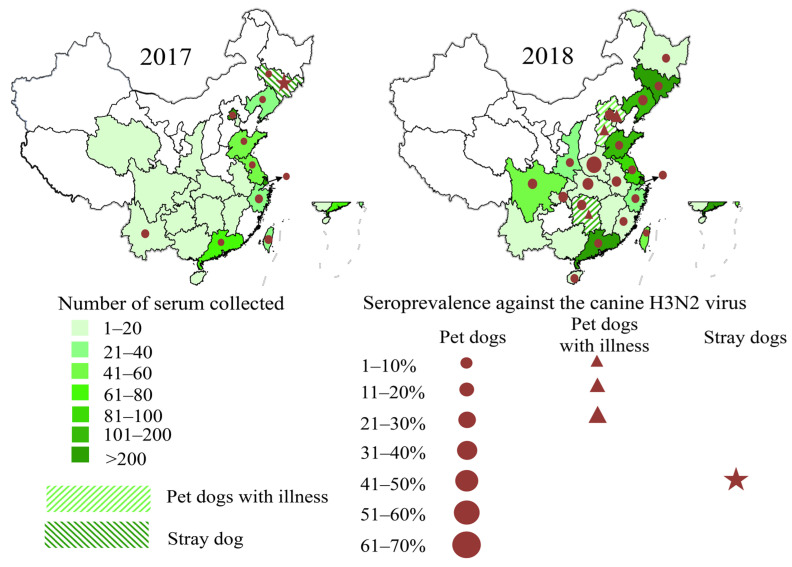
Prevalence rates of H3N2 CIVs in dogs across China in 2017–2018. (**a**) Prevalence rates of H3N2 CIVs in dogs across China in 2017. (**b**) Prevalence rates of H3N2 CIVs in dogs across China in 2018. Provinces with sampling points are marked green, and the sampling quantity is reflected by the color depth. Red dots, samples from pet dogs; red triangle and stars, samples from pet dogs with illness and stray dogs, respectively. The symbol size was used to describe the size of seroprevalence.

A total of 3579 dog serum samples from 27 provinces in China were screened by the HI assay for antibody reactivity against A/canine/Jilin/QiaoKe01/2017 (QK01(H3N2)). As shown in Table 1 and Figure 2, 528 (14.75%) dog serum samples were positive. The results demonstrated prevalence rates of 6.68% (61/913) to 48.46% (63/130) among dogs with varied living conditions. The seroprevalence rates of canine H3N2 influenza virus in pet dogs were 6.68% and 18.89% in 2017 and 2018, respectively, while stray dogs showed a rate of 48.46% in 2017. Furthermore, serum samples from dogs with respiratory symptoms were positive for canine H3N2 influenza virus at a rate of 19.71%. These findings indicated that the canine H3N2 influenza virus spread rapidly in dogs after entering China in 2016.

### 3.4. Replication and Transmission of Canine H3N2 Influenza Virus in Beagles

**Figure 3:**
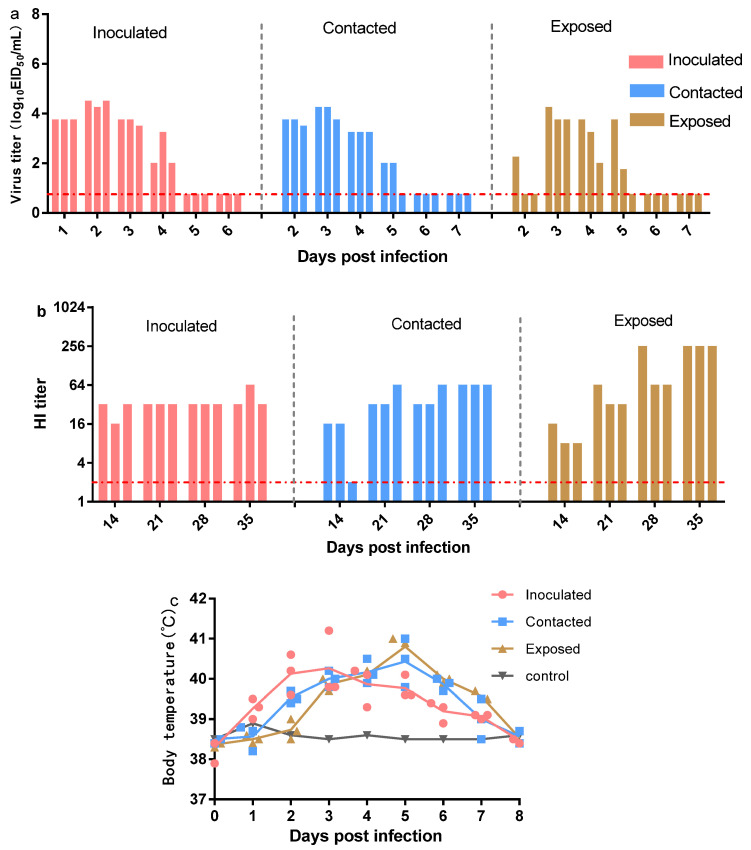
Transmission of canine H3N2 CIVs in beagles. (**a**) The vertical axis represents the titers of influenza viruses recovered from nasal washes of virus-inoculated, contacted, and exposed dogs. The dashed line represents the limit of detection. (**b**) HI antibody titers of the tested animals. Each column represents an individual animal. The dashed line represents the limit of detection. (**c**) Changes in body temperature after canine H3N2 infection.

The replication and transmission of the virus in beagles were assessed in the contact and exposed groups at 1 dpi and thereafter. The results showed that beagles in the inoculated, contact, and exposed groups had typical respiratory symptoms, including sneezing, watery eyes, coughing, and shortness of breath. As shown in Figure 3, the rectal temperature of beagles in all groups, except the control group, was raised (39.0–41.1 °C). The nasal and throat swabs of each dog were mixed to determine the EID_50_ of chicken embryos, and infectious virus particles could be detected in beagles in all groups except the control group.

Furthermore, HI antibodies in each dog were detected at 14, 21, 28, and 35 dpi. At 14 dpi, HI antibodies were observed in the inoculation (3/3), contact (2/3), and exposed (3/3) groups. At 21 dpi, all experimental dogs showed high HI antibody titers (32–64). At 35 dpi, the exposed group (256) showed higher HI antibody titers than the inoculated (32–64) and contact (64) groups. These results suggested that natural infection with H3N2 CIV may contribute to the expansion of memory B cells, enabling the production of more antibodies. The above findings indicated that the H3N2 CIV could efficiently be transmitted via contact and respiratory droplets.

**Figure 4:**
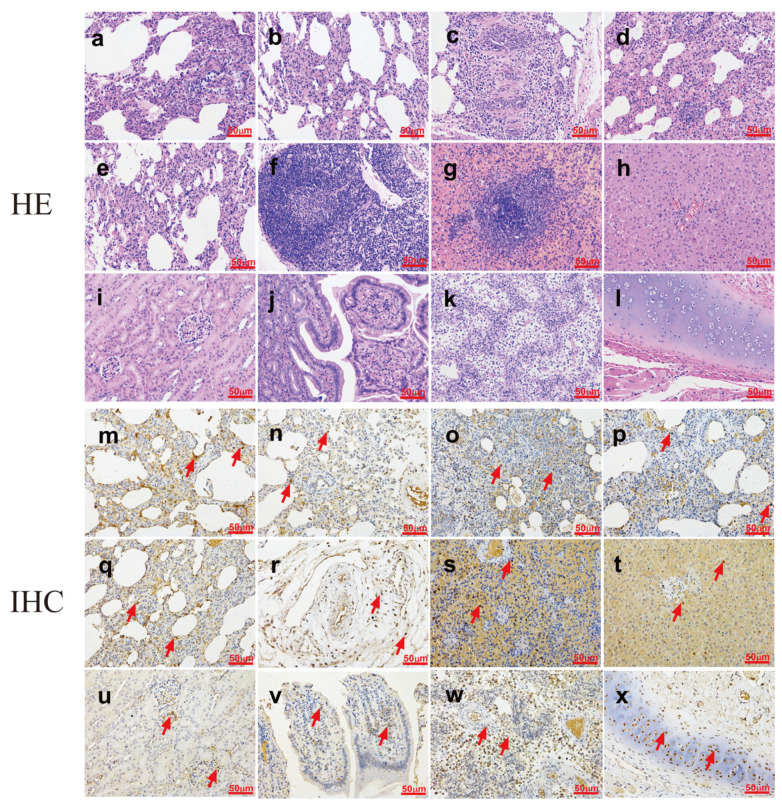
Histopathological analysis of dogs. Dogs were killed on 3 dpi with 10^7^ EID_50_ of the test virus. H&E staining images (**a**–**l**). (**a**–**e**) Lungs from H3N2 virus-infected dogs, with collapsed alveolar septum and alveolar cavity expansion and some lymphocyte infiltration of the stroma. (**f**) Hilar lymph nodes in dogs infected with H3N2 virus manifested as lymphoid tissue hyperplasia. (**g**) The liver of dogs infected with the H3N2 virus shows mild edema of hepatocytes. Spleen (**h**), kidney (**i**), and duodenum (**j**) are normal. (**k**) The mesenteric lymph node tissues of H3N2 virus-inoculated animals show mild histopathological changes. (**l**) Sloughing in the trachea. IHC for nucleoprotein (NP) detection in sections of intranasally infected dogs. Images from (**m**) to (**x**) after IHC: (**m–q**) a high viral antigen titer was detected in the lung tissues. Hilar lymph nodes (**r**), liver (**s**), spleen (**t**), kidney (**u**), duodenum (**v**), mesenteric lymph nodes (**w**), and trachea (**x**) showed only a mild presence of viral antigen. Red arrow, viral antigen positivity. Scale bars in (**a**–**x**), 50 μm.

Subsequently, histological assays were conducted to assess pathological changes in virus-infected dogs at 3 dpi (Figure 4). H&E results showed collapsed alveolar septum, expanded alveolar cavity, and a small number of lymphocytes infiltrated in the interstitium. All lung lobes showed a collapsed alveolar compartment, expanded alveolar cavity, and a small amount of lymphocyte infiltration in the interstitium. The trachea showed shedding of the mucosal epithelium, and hilar lymph nodes had lymphoid tissue hyperplasia. No abnormalities were observed in the spleen, kidneys, and intestines, but the liver showed mild edema. In addition, mesenteric lymph nodes showed mild hyperplasia of the lymphatic tissue. IHC results demonstrated large amounts of viruses in the lungs (upper left, lower left, middle, upper right, and lower right), pulmonary lymph nodes, liver, spleen, kidney, intestine, mesenteric lymph nodes, and tracheal tissue, while liver and kidney carried a small number of viruses post-infection. These findings suggested that canine H3N2 CIV could lead to multi-tissue infection in the host and pathological damage in dogs.

### 3.5. Pathogenesis in Mice

**Figure 5:**
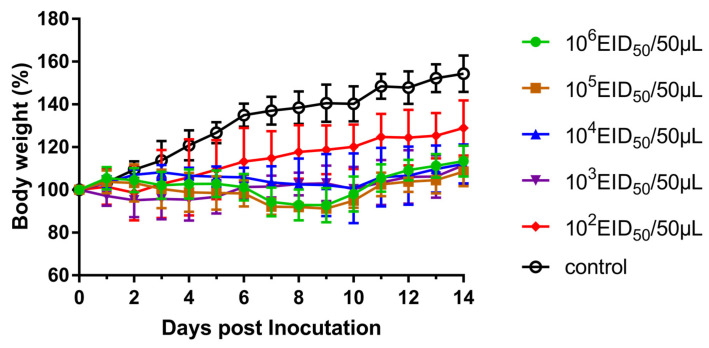
Morbidity rate of the new clade (represented by qk01/2017) CIV in BALB/c mice. Percentages of the animal’s weight change on the day of inoculation.

**Figure 6:**
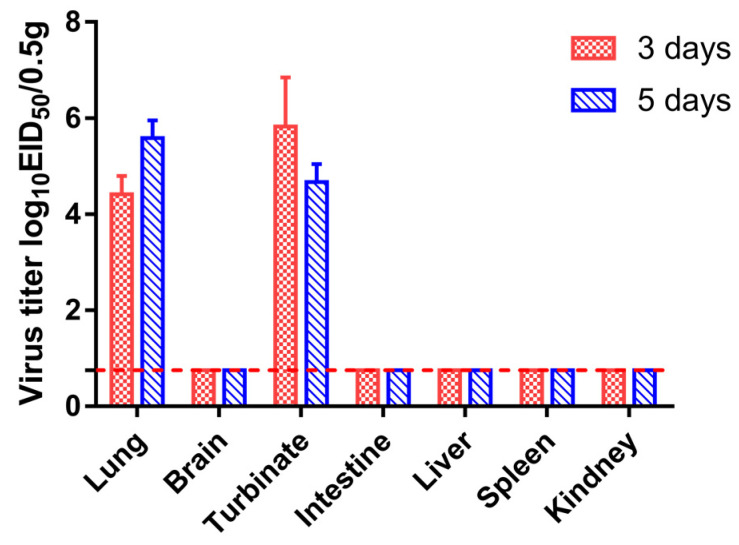
Tissue tropism of the new clade (represented by qk01/2017) CIV. Mice (*n* = 3) were infected with 10^6^ EID_50_ of the qk01/2017 isolate. Lung, nasal turbinate, brain, liver, spleen, kidney, and intestine samples were collected, and viral titers were determined at 3 and 5 dpi. The dotted line indicates the detection limit.

To evaluate the replication of viruses in mammals, we inoculated groups of five mice i.n. with five diluted solutions 10^2^–10^6^ EID_50_ of the virus, respectively. As shown in Figure 5, mice infected with the influenza virus had a slower weight gain than the control group. Furthermore, mice infected with 10^6^ EID_50_ of influenza virus were killed on 3 and 5 dpi, and their organs, including nasal turbinate, lung, spleen, kidney, and brain, were harvested for viral titer detection. Viral replication was detected in the nasal turbinate and lung of mice but not in the spleen, kidney, or brain (Figure 6). These results indicated that the H3N2 CIV could replicate in the respiratory tract of mice.

### 3.6. Replication and Transmission in Guinea Pigs

**Figure 7:**
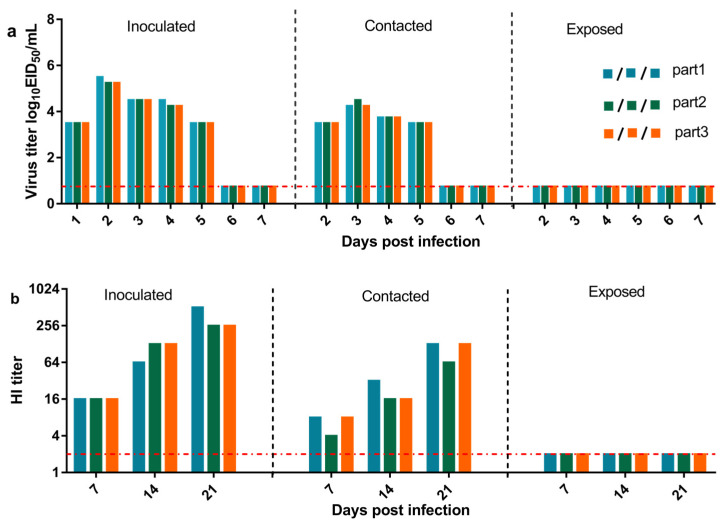
Replication and transmission in guinea pigs of H3N2 CIVs. (**a**) The vertical axis represents the titers of influenza viruses recovered from nasal washes in virus-inoculated, contacted, and exposed guinea pigs. (**b**) HI antibody titers of the animals. Each color bar represents an individual animal. The dashed line represents the limit of detection.

Guinea pigs have been used as animal models to evaluate the replication and transmission of the influenza virus. Nasal washes and serum samples were obtained to detect the virus and HI antibody titers. As shown in Figure 7, guinea pigs in the inoculated and contact groups had high viral titers in nasal washes at 2–5 dpi, while the exposed group showed no viral shedding at any time point. Serum samples from guinea pigs in the inoculated and contact groups were positive at 7, 14, and 21 dpi, while the exposed group was negative. The above data indicated that the H3N2 CIV is transmitted by contact and not by respiratory droplets.

### 3.7. Infection of Chickens and Ducks

**Figure 8:**
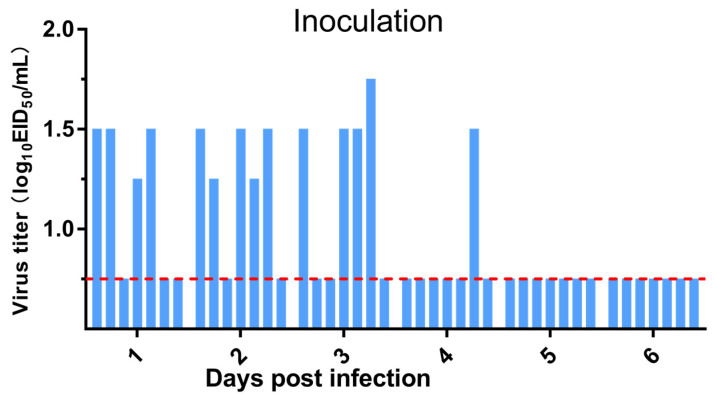
Replication in chickens of H3N2 CIVs. Viral titers in throat and cloacal swabs mixed samples after infection with H3N2 CIVs. A group of seven chickens was inoculated with 3 × 10^6^ EID_50_/0.1 mL of the test virus. Each blue bar represents the virus titer in an individual animal. The dashed red line indicates the lower limit of detection.

Furthermore, SPF chickens and domestic ducks were chosen to assess whether the virus could infect poultry after long-term adaptation in dogs. As shown in Figure 8, low levels of virus replication could be detected in the throat and cloacal swabs of chickens in the inoculated group by the chicken embryo infection titration assay at 1–4 dpi, while none was detected in the contact and exposed groups. Moreover, serum antibody levels in chickens were monitored by the HI titer assay at 7, 14, and 21 dpi. The results showed that HI titers in the infected group were 32–64 on 14 dpi and increased to 1:128 on 21 dpi. The contact and exposed groups were negative(Table 2). Furthermore, virus replication in the throat and cloacal swabs and HI titers in serum could not be detected in ducks of the inoculated, contact, or exposed group. The above data indicated that the H3N2 CIV could only replicate inefficiently in chickens and could not infect ducks.

## 4. Discussion

H3N2 CIV, a pathogen that causes severe and acute respiratory disease in dogs, has been circulating in China for more than a decade. However, the prevalence and outbreak mode of this virus remains to be further studied. The emergence of the H3N2 CIV and its evolutionary and epidemiological patterns with multiple, periodic introductions in the USA were described by Voorhees et al. [25]. In the present study, we reported the isolation of H3N2 CIVs genetically related to strains identified in dog populations in the USA in 2017, indicating an incursion into China from the USA. This revealed that the recent circulation of the H3N2 CIV formed a separate clade, distinct from other isolates of China before 2017. No virus belonging to the clade known before 2016 was detected, suggesting that the new antigenic variation lineage occurred in 2017 and has been dominant in the dog population. The underlying evolutionary drivers need to be further examined.

Animal studies demonstrated that elevated body temperature, virus shedding, and seroconversion were detected in infected and contacted beagles. Experimental infections in mice, guinea pigs, and beagles provided insights into the pathogenicity of these viruses in mammals. Mild histopathological changes, including hyperplasia of pneumocytes and infiltration of lymphocytes, were recorded in guinea pigs and beagles infected with the QK01 isolate. Furthermore, the transmission experiment showed that the QK01 isolate could be transmitted to naïve beagles via respiratory droplets, which suggested that currently circulating strains might be readily transmitted among mammalian hosts. Dog-to-dog transmission through respiratory droplets of the circulating canine virus might cause a rapid spread of the virus in a short period in the dog population. Considering the large number of pet dogs and the increased probability of contact between humans and pets, there is a potential risk of virus transmission from dogs to humans.

Taken together, these results suggested that currently circulating H3N2 CIVs antigenic variants might have entered China from the USA. Furthermore, these viruses are continuously evolving in canine species, have expanded the host range, and might be established in pet cats. Importantly, these findings underscore the importance of continuous surveillance to understand the genetic diversity and zoonotic risk of CIVs in China.

## Figures and Tables

**Table 1:** Seroprevalence of CIV in pet dogs in China.

Sample Source	Number of Samples	Positive Number	Positive Rate (%)	HI Titer		
				8–32	64–256	512–1024
Pet dogs (2017)	913	61	6.68	33	25	3
Stray dogs (2017)	130	63	48.46	51	12	0
Pet dogs (2018)	2284	363	18.89	117	200	46
Dogs with respiratory symptoms (2018)	208	41	19.71	17	19	5
Chinese rural dogs (2018)	44	0	0	-	-	-
Total	3579	528	14.75	218	256	54

**Table 2:** Seroconversion of SPF chickens and ducks after H3N2 CIV infection.

Group	Day	Seroconversion (HI Antibody Titer Range)			
		Inoculated	Contacted	Exposed	Control
Chicken	7	0/7	0/3	0/3	0/3
	14	3/7 (64, 64, 32)	0/3	0/3	0/3
	21	3/7 (128, 128, 128)	0/3	0/3	0/3
Duck	7	0/3	0/3	0/3	0/3
	14	0/3	0/3	0/3	0/3
	21	0/3	0/3	0/3	0/3 ^1^

^1.^ Data are presented as the number of seropositive animals divided by that of inoculated/contacted or exposed animals. The antibody titers of HI are stated in parentheses.

## Data Availability

The gene sequences used in this study are openly available on GenBank.

## Supplementary Materials

The following are available online at https://www.mdpi.com/article/10.3390/v13112279/s1, Table S1. Primer of 8 non-coding regions gene fragment sequence of H3N2 CIV. Figure S1. Diagram of H3N2 CIV infection in guinea pigs. Supplementary Figure S2. Phylogenetic tree of H3N2 CIV internal sequences.
