# Supplementary material for: Characterization of Canine Influenza Virus A (H3N2) Circulating in Dogs in China from 2016 to 2018
Source: Viruses. 2021 Nov 15;13(11):2279. doi: 10.3390/v13112279 (PMC8618230; doi:10.3390/v13112279)
Supplement: Supplementary file 1 [file viruses-13-02279-s001.zip › viruses-1420202-supplementary.pdf]

viruses

Supplementary Materials

Characterization of Canine Influenza Virus A (H3N2) Circulating in Dogs in China from 2016 to 2018

Table S1. Primer of 8 non-coding regions gene fragment sequence of H3N2 CIV.

Primers	Sequences (3′–5′)
PB2	F:TAAGAGATCTAATGTCGCAGTC<br>R:TGTCAGTAAGTATGCTAGAG
PB1	F:GTCATCCGACTTTACTTTTCTTGAAGTGCC<br>R:GGAGTTTTTTCATGAAGGACAAGCT'
PA	F:GCTTCAATCCAATGATTGTCGAGCTTG<br>R:GTAGCATTGCCACAACTATTTCAGTGCATG
HA	F:GCAAAAGCAGGGGATACTTTCATTAATCAT<br>R:CACATAATGAAACCCAGCAAGACAACA
NP	F:AACGATCGTATGAACAGATGGAAACTG'<br>R:TCTTCTGCATTGTCTCCGAAGAAATAGGAT
NA	F:TAATAGCAATAGGGTCTGTCTCTCTAGCCAT<br>R:TATAGGCATGAAGTTAATATTCGCCCCATC
M	F:TATTGAAAGATGAGTCTTCTAACCGAGGTC<br>R:GGTAGTTTTTTACTCCAGCTCTATGTTGAC
NS	F:AACATAATGGACTCCAACACTGTGTCAAG<br>R:AGCTGAAACGAGAAAGTTCTTATCTCTTGC[1]

[1]The primers included an outer forward primer (F), an outer backward primer (R).

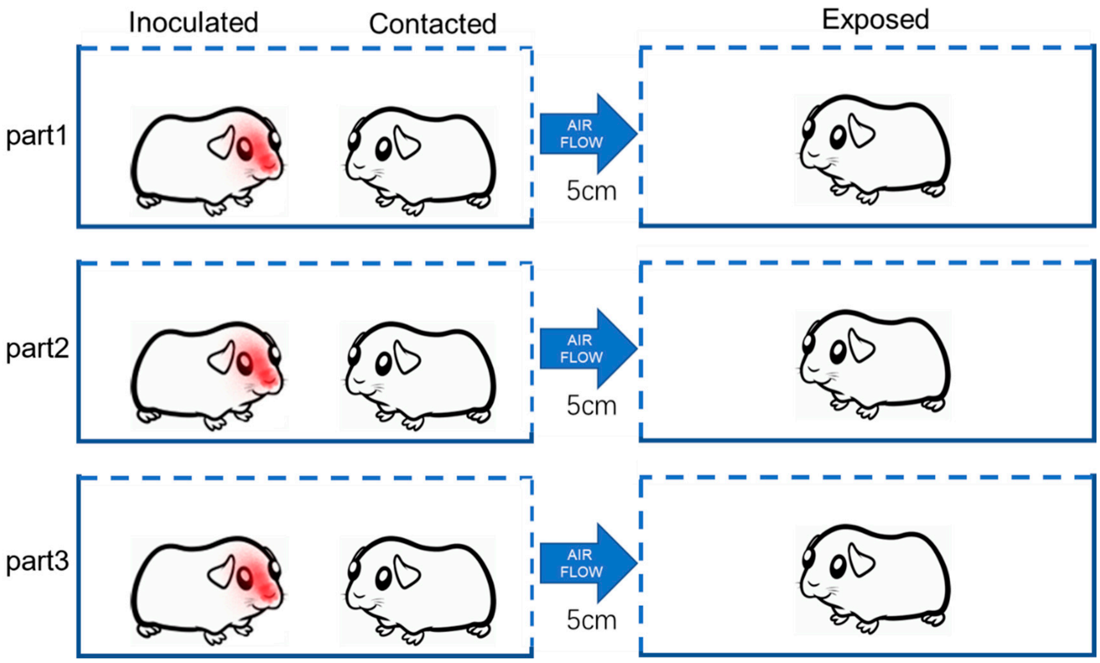

Figure S1. Diagram of H3N2 CIV infection in guinea pigs. Three animals were inoculated with 2×10^6 EID_{50} of H3N2 CIV. At 24 h p.i., each of these animals was placed in a cage with one uninfected guinea pig. Another one uninfected guinea pig was placed in a nearby cage (5cm apart). Arrows indicate the direction of air flow. Cage layouts are shown in Diagram. Nasal washes were collected once daily, beginning on day 2 p.i. (1 day post-exposure).

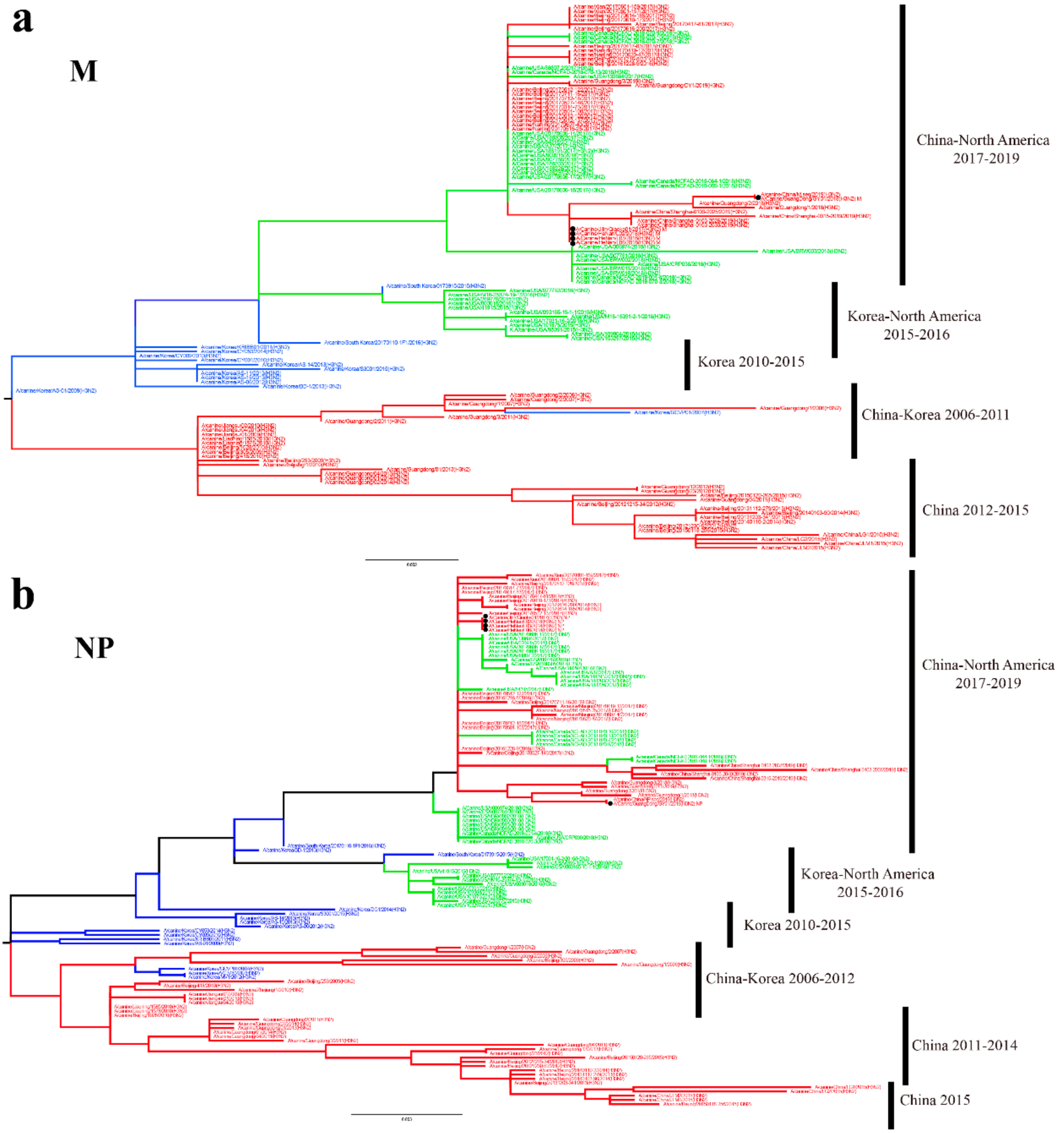
a
M
China-North America 2017-2019
Korea-North America 2015-2016
Korea 2010-2015
China-Korea 2006-2011
China 2012-2015
b
NP
China-North America 2017-2019
Korea-North America 2015-2016
Korea 2010-2015
China-Korea 2006-2012
China 2011-2014
China 2015

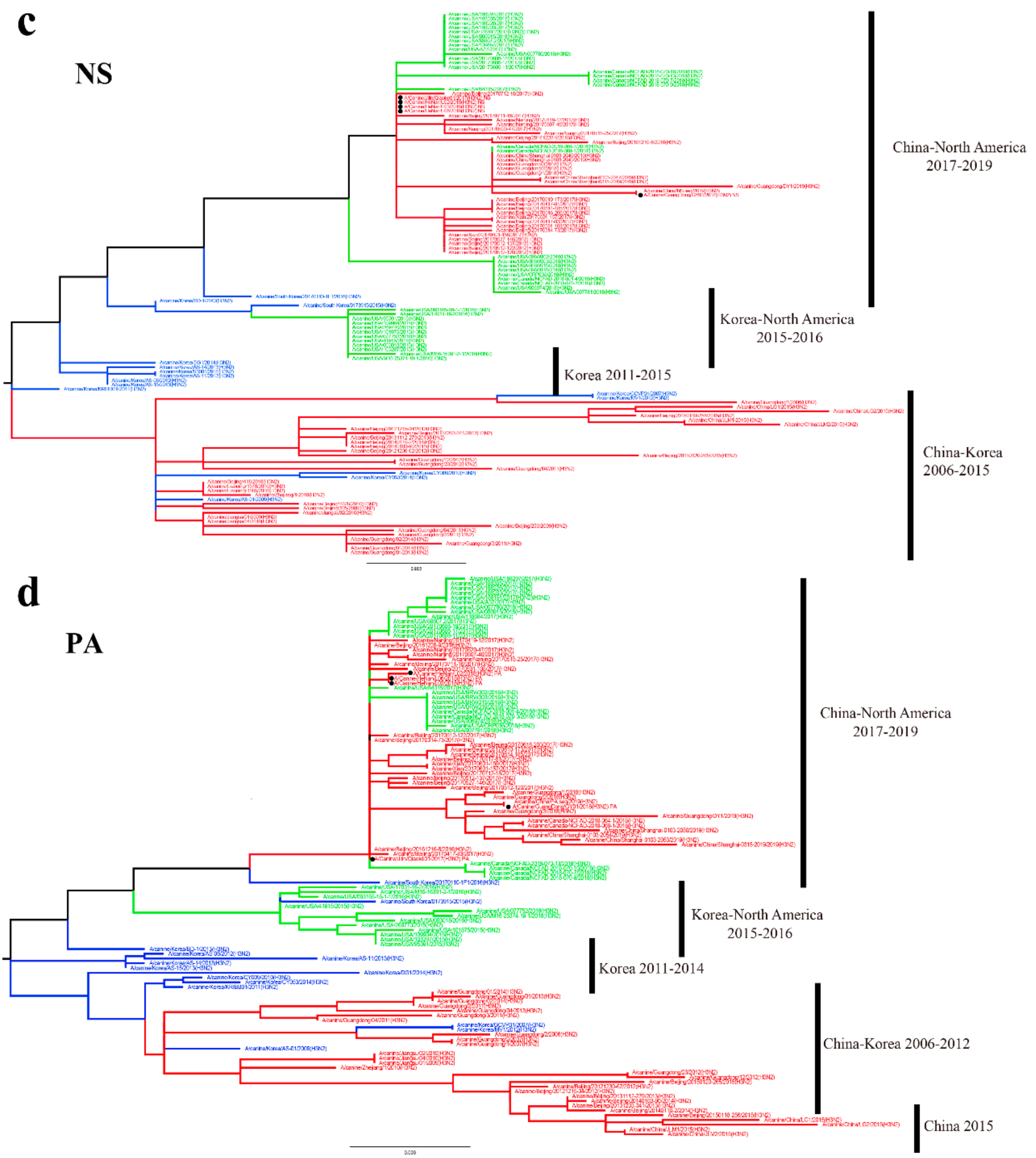
c
NS
China-North America 2017-2019
Korea-North America 2015-2016
Korea 2011-2015
China-Korea 2006-2015
d
PA
China-North America 2017-2019
Korea-North America 2015-2016
Korea 2011-2014
China-Korea 2006-2012
China 2015

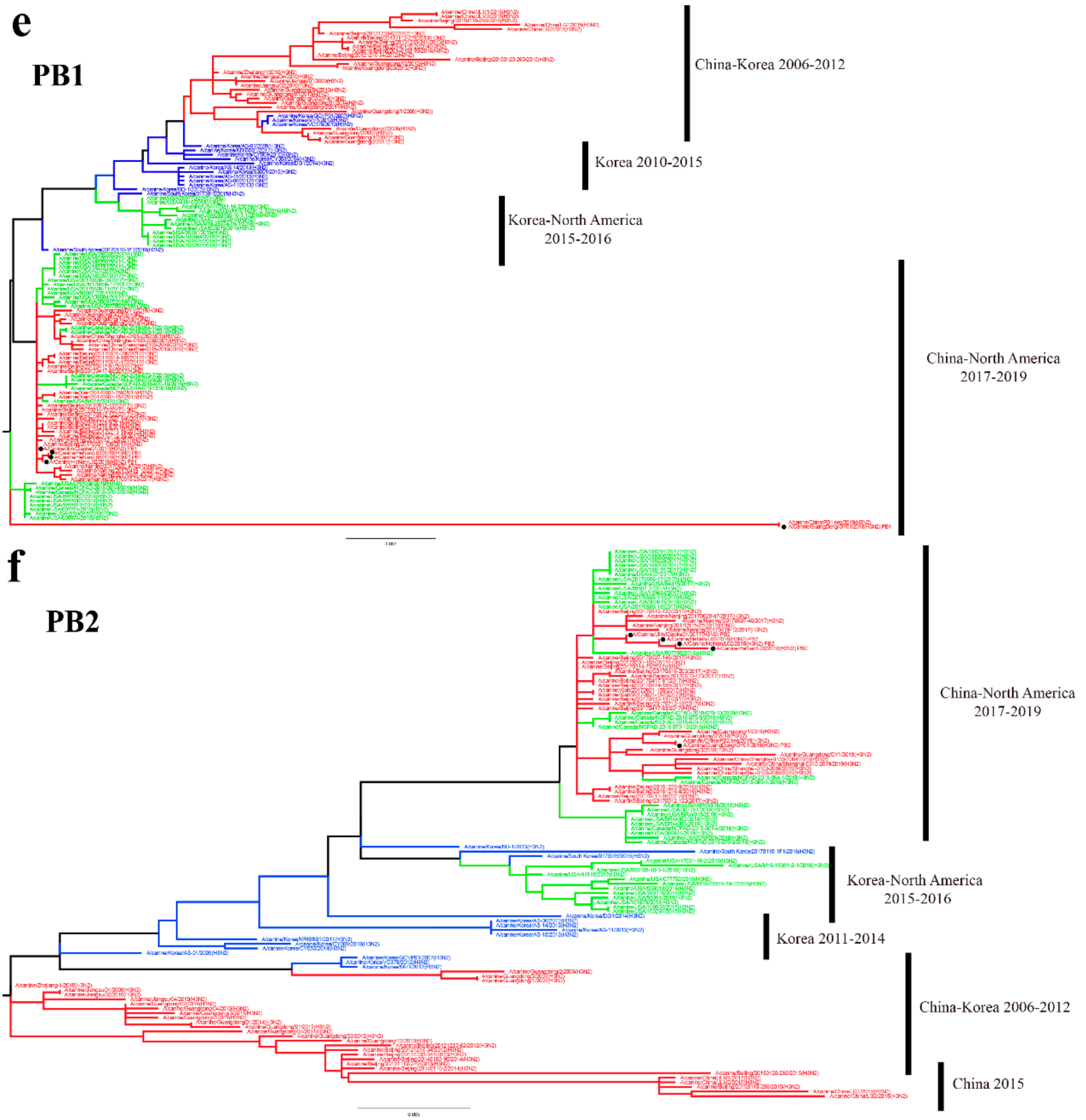

Figure S2. Phylogenetic tree of H3N2 CIV internal sequences. Phylogenetic trees based on the nucleotide sequences of M (a), NP (b), NS (c), PA (d), PB1 (e), PB2 (f) genes. In all the trees, green branches on the phylogenies represent viruses isolated in North America, red branches represent those isolated in China, and blue branches represent those isolated in South Korea. The black circle represents the strains in this study.
